# Characterization and Expression Analysis of the *PvTLP* Gene Family in the Common Bean (*Phaseolus vulgaris*) in Response to Salt and Drought Stresses

**DOI:** 10.3390/ijms26125702

**Published:** 2025-06-13

**Authors:** Xue Dong, Min Zhao, Jia Li, Fuyi Qiu, Yan Wang, Jiandong Zhao, Jianwu Chang, Xiaopeng Hao

**Affiliations:** 1 Center for Agricultural Genetic Resources Research, Shanxi Agricultural University, Taiyuan 030031, China; jodiedong@sxau.edu.cn (X.D.); wangyan204408@sxau.edu.cn (Y.W.); zhaojiandong@sxau.edu.cn (J.Z.); changjianw2005@163.com (J.C.); 2 Key Laboratory of Crop Gene Resources and Germplasm Enhancement on Loess Plateau, Ministry of Agriculture, Taiyuan 030031, China; 3 Shanxi HouJi Laboratory, Shanxi Agricultural University, Taiyuan 030031, China; 4 College of Agriculture, Shanxi Agricultural University, Taiyuan 030031, China; 20232165@stu.sxau.edu.cn (M.Z.); z20223131@stu.sxau.edu.cn (J.L.); 20233231@stu.sxau.edu.cn (F.Q.)

**Keywords:** tubby-like proteins (TLPs), *Phaseolus vulgaris*, salt and drought stresses, expression profile, qRT-PCR

## Abstract

Tubby-like proteins (TLPs) are essential multifunctional transcription factors in plants that significantly influence plant growth and development, signal transduction, and adaptation to environmental stress. Despite their importance, there is limited knowledge of the identification and functional roles of the *TLP* gene family in the common bean. In this study, we identified the *PvTLP* gene family, which consists of 10 *PvTLP* genes distributed unevenly across seven chromosomes. Phylogenetic analysis revealed that these genes could be classified into three subfamilies (A, B, and C). All PvTLP proteins contained both conserved tubby and F-box domains, with the exception of PvTLP7, which lacks the F-box domain. Conserved motif analysis revealed that 10 *PvTLP* genes contained motif 1 and motif 3. Cis-acting elements analysis indicated that *PvTLP* genes might be involved in light, hormone, and stress responses. Synteny analysis revealed a closer phylogenetic relationship between the common bean and dicotyledons than monocotyledons. qRT-PCR analysis confirmed the significant differences in the expression of most *PvTLP* genes in both leaves and roots under salt and drought stresses. These findings provide valuable insights for further exploration of the molecular functions of TLPs in plant responses to various stresses and offer key candidate genes for enhancing stress resistance in the common bean through molecular breeding.

## 1. Introduction

Tubby-like proteins (TLPs) are multifunctional transcription factors that are widespread across eukaryotic organisms [1]. The *TLP* gene was first observed in obese tubby mice [2]. In mammals, mutations in *TLP* genes may lead to various diseases, including hearing loss, retinal degeneration, and obesity [3]. TLPs are characterized by their conserved tubby domain at the carboxyl terminus [4,5]. This domain demonstrates that a central hydrophobic α-helix traverses through a closed β-barrel composed of 12 antiparallel strands [6]. TLPs are anchored to the plasma membrane through interactions with phosphatidylinositol 4,5-bisphosphate (PI(4,5)P2) [7,8]. Activation of G-protein signaling cascades triggers the translocation of TLPs to the nucleus, where they are involved in G-protein-coupled receptor-mediated signal transduction [9,10]. In plants, in addition to the tubby domain at the carboxyl terminus, most TLP proteins harbor a conserved F-box domain in the amino-terminal region that serves as a component of the Skp1-Cullin-F-box (SCF) E3 ubiquitin ligase complex [11]. The F-box domain facilitates participation in ubiquitination pathways through substrate recognition and recruitment to SCF, thereby regulating the SCF-mediated ubiquitin proteolytic pathway [12,13].

More members of the *TLP* gene family have been identified in plants than in animals, including *Arabidopsis thaliana* (11), *Oryza sativa* (14), *Gossypium hirsutum* (12), *Glycine max* (20), *Solanum lycopersicum* (11), and *Malus domestica* (9) [14,15,16,17,18,19]. TLPs have been suggested to be molecular regulators of plant growth, signal transduction cascades, and adaptive responses to hormone and abiotic stresses. Strawberry *FvTLPs* exhibited dynamic responsiveness to salinity, drought, cold, and hormone treatments [20]. In cassava, *MeTLPs* were found to respond to salt, drought, and low-temperature stresses [21]. *Arabidopsis* AtTLP2 serves as a critical modulator of ABA signaling and dehydration responses, with *AtTLP2*-overexpressing lines exhibiting markedly increased tolerance to drought and salinity during maturation phases. In contrast, the *attlp2* mutant was found to display sensitivity to ionic, osmotic, and oxidative stresses [22]. *AtTLP2* exerted positive regulatory control over UDP-glucose 4-epimerase, modulating homogalacturonan biosynthesis in seed coat mucilage through an ABA-dependent pathway [23]. AtTLP3 and AtTLP9 share high homology. Single mutants of *attlp3* or *attlp9* were found to exhibit tolerance to ABA and osmotic stresses during seed germination and early developmental stages [14,24]. The *attlp3*/*attlp9* double mutant presented a higher germination frequency than the single mutant under ABA and osmotic stresses, suggesting that *AtTLP3* has a redundant function with *AtTLP9* in *Arabidopsis* [24]. In chickpea, CaTLP1 might interact with protein kinases to regulate ABA-dependent gene transcription and stomatal dynamics, thereby conferring enhanced drought adaptation ability [25,26]. Cucumber CsTLP8 functions as a negative regulator of osmotic stress and ABA signaling by affecting antioxidant enzymatic activity, consequently impairing seed germination and seedling establishment while increasing ABA hypersensitivity [27].

The common bean (*Phaseolus vulgaris* L.) is a globally cultivated legume of critical agricultural importance. It plays a crucial role in maintaining dietary balance and enhancing human nutrition owing to its abundance of plant-based proteins, essential amino acids, dietary fiber, and vitamins [28,29,30]. However, the growth and yield potential of this legume are often restricted by various abiotic stresses, which severely limit its productivity [31,32]. Consequently, understanding the molecular mechanisms that regulate common bean responses to abiotic stress is essential for developing cultivars that are tolerant to salt and drought stresses, ultimately improving crop yields. Although the *TLP* gene family has been widely characterized in various plants, its role in the common bean remains poorly understood. This research aimed to comprehend the evolutionary history and the abiotic stress response of *PvTLP* genes.

In this study, a comprehensive genome-wide identification of the *PvTLP* gene family in the common bean was performed, and the physicochemical properties, chromosomal distribution and gene duplication, evolutionary relationship, gene structure, conserved motif, synteny, and cis-acting element analyses were determined. Additionally, tissue and abiotic stress-induced expression profiles of *PvTLP* genes were analyzed. The results provide theoretical insights into the stress-responsive molecular mechanisms of *PvTLP* genes and offer a new perspective for resistance breeding in the common bean.

## 2. Results

### 2.1. Identification and Characterization of the PvTLP Gene Family

A local BLASTP search was performed using AtTLPs as references, along with HMMER screening targeting the conserved tubby domain (PF01167). This study identified 10 members of the *PvTLP* gene family in the genome of the common bean. These genes were designated *PvTLP1*–*PvTLP10* according to their chromosomal positions. Detailed information on their predicted physiological properties is listed in Table 1. The CDS lengths of *PvTLP* genes ranged from 1083 bp (*PvTLP3*) to 1296 bp (*PvTLP10*). The encoded protein lengths varied from 360 to 431 amino acid residues, with molecular weights (MWs) ranging from 40,469.27 to 48,120.22 Da. The predicted isoelectric points (pI) were between 9.09 and 9.71, with an average of 9.42, indicating that the PvTLP proteins had alkaline properties.

In addition to PvTLP7, which exhibited a lower instability index of 37.45, the scores of the other PvTLPs exceeded 50, indicating relative instability. The aliphatic index varied from 65.62 (PvTLP7) to 80.93 (PvTLP1), and all PvTLPs presented negative GRAVY values, confirming their hydrophilic characteristics. Subcellular localization predictions revealed that seven PvTLPs were localized in the nucleus, two in the mitochondria, and one in the chloroplast. These findings provide fundamental data for investigating the evolution and function of the *PvTLP* gene family.

### 2.2. Chromosomal Localization and Gene Replication Events of the PvTLP Gene Family

**Figure 1:**
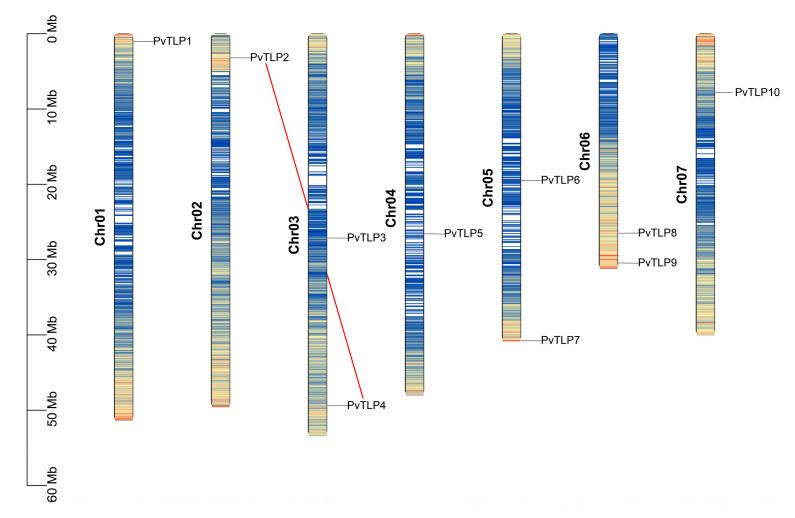
Chromosomal localization and segmental duplication events of *PvTLP* genes. The segmentally duplicated gene pair is marked with a red line.

Chromosomal mapping analysis revealed that the 10 *PvTLP* genes were distributed on 7 of the 11 chromosomes (Figure 1). Chr03, Chr05, and Chr06 each contained two genes, while Chr01, Chr02, Chr04, and Chr07 each possessed one gene. No *PvTLP* genes were identified on Chr08 to Chr11. This chromosomal distribution pattern reveals the uneven and irregular spatial characteristics of the *PvTLP* gene family.

To elucidate the evolutionary expansion and functional diversification of the *PvTLP* gene family, we examined gene duplication events. The *PvTLP* gene family contained one segmentally duplicated gene pair (*PvTLP2*/*PvTLP4*) and no tandem duplication. Further, the non-synonymous and synonymous substitution ratio (Ka/Ks ratio) was calculated for the segmentally duplicated *PvTLP* gene pair to assess the selection pressure during evolution (Supplementary Table S1). The Ka/Ks ratio was less than 1, indicating that the expansion of *PvTLP* genes has undergone purifying selection pressure during evolution.

### 2.3. Phylogenetic Analysis of the PvTLP Gene Family

**Figure 2:**
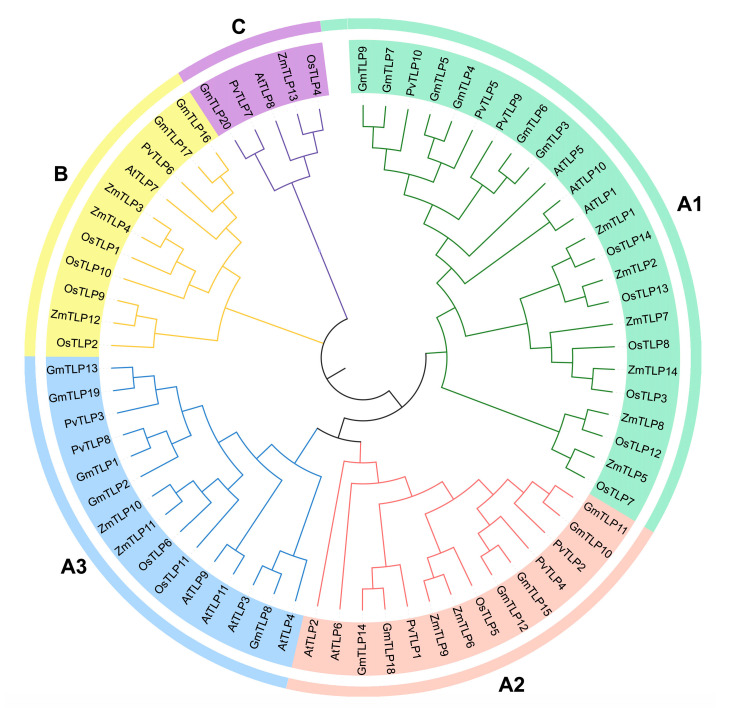
Phylogenic tree of TLP proteins from *Phaseolus vulgaris*, *Arabidopsis thaliana*, *Glycine max*, *Oryza sativa*, and *Zea mays.* A phylogenetic tree was constructed using the neighbor-joining method with 1000 bootstrap replicates. TLP proteins were categorized into three subfamilies (A–C), with subfamily A further clustered into three subgroups (A1–A3).

To elucidate the evolutionary relationships among TLP proteins across various plant species, multiple sequence alignment of full-length TLP protein sequences from the common bean (10), *Arabidopsis* (11), rice (14), maize (14), and soybean (20) was conducted using Muscle (Supplementary Table S2). Phylogenetic analysis was performed with the MEGA11 software. The neighbor-joining (NJ) phylogenetic tree revealed that 69 TLP proteins were categorized into three subfamilies (A, B, and C, Figure 2). Subfamily A, the largest group, containing 53 TLP members, was further clustered into three distinct subgroups (A1, A2, and A3). Subgroup A1 had 24 TLP proteins, including 3 PvTLPs (PvTLP5, PvTLP9, PvTLP10), 3 AtTLPs, 6 GmTLPs, 6 OsTLPs, and 6 ZmTLPs. Subgroup A2 had 14 TLP proteins, which encompassed 3 PvTLPs (PvTLP1, PvTLP2, PvTLP4), 2 AtTLPs, 6 GmTLPs, 1 OsTLP, and 2 ZmTLPs. Subgroup A3 had 15 TLP proteins, comprising 2 PvTLPs (PvTLP3, PvTLP8), 4 AtTLPs, 5 GmTLPs, 2 OsTLPs, and 2 ZmTLPs. Subfamily B, containing 11 TLP proteins, consisted of 1 PvTLP (PvTLP6), 1 AtTLP, 2 GmTLPs, 4 OsTLPs, and 3 ZmTLPs. Subfamily C was the smallest group, with only one member from each species, including PvTLP7, AtTLP8, GmTLP20, OsTLP4, and ZmTLP13. PvTLPs and GmTLPs exhibited a distinct clustering pattern, with nine PvTLPs showing a 1:2 homologous relationship with GmTLPs. For instance, PvTLP3 paired with GmTLP13/GmTLP19, PvTLP4 with GmTLP12/GmTLP15, and PvTLP6 with GmTLP16/GmTLP17. These findings indicate that the TLPs of the common bean and soybean are significantly more homologous than those of other species.

### 2.4. Gene Structure, Conserved Domain, and Motif Analysis of the PvTLP Gene Family

**Figure 3:**
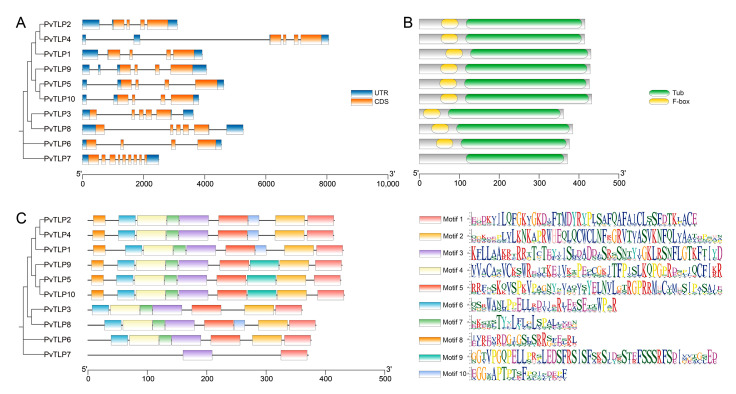
Gene structure, conserved domain, and motif analysis of the *PvTLP* gene family: (**A**) gene structure of *PvTLP* genes; (**B**) conserved domains of PvTLP proteins; (**C**) conserved motifs of PvTLP proteins and the amino acid consensus sequence logos of these motifs.

Gene structure serves as crucial evidence for elucidating phylogenetic relationships. By utilizing the coding sequences and genomic annotations of *PvTLP* genes, the number and distribution of exons and introns across *PvTLP* genes were predicted. The members of subgroup A1, subgroup A2, and subfamily B contained three introns; subgroup A3 contained four introns; and subfamily C contained eight introns (Figure 3A). These results indicate that *PvTLP* genes present similar gene structures.

Conserved domain analysis revealed that subfamilies A and B contained both conserved tubby and F-box domains, except subfamily C, which had a single tubby domain (Figure 3B). Based on the MEME suite, ten conserved motifs in PvTLPs were identified, and some differences in the distribution of the motifs were observed (Figure 3C). All of the PvTLP proteins contained motif 1 and motif 3, which belong to the tubby domain and likely contribute to the functional conservation of TLP proteins. Subfamilies A and B shared motif 2, motif 4, motif 5, motif 6, and motif 7. Among the subfamily A members, motif 9 was found only in subgroup A1, and motif 8 appeared in both subgroups A1 and A2. These findings suggest that PvTLP proteins in the different subfamilies contain various conserved motifs, which result in functional diversity.

### 2.5. Synteny Analysis of TLP Genes

**Figure 4:**
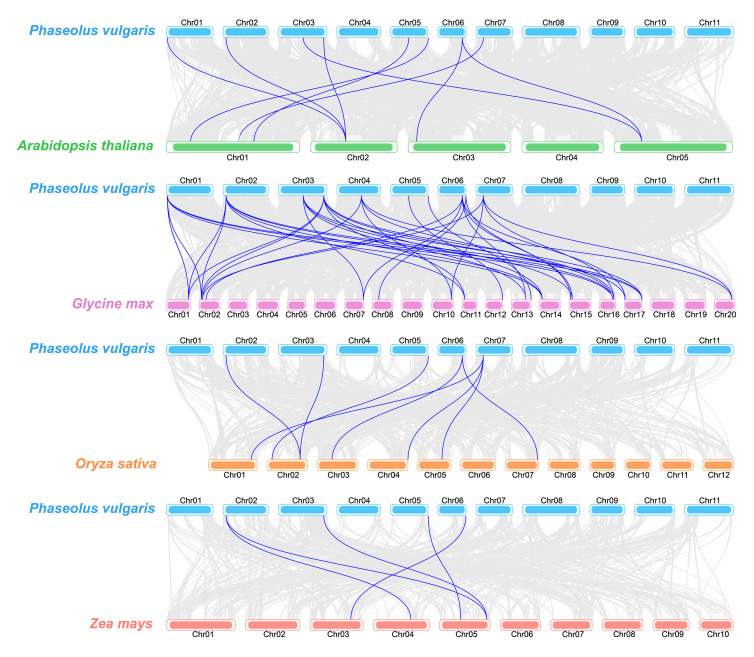
Synteny analysis of *TLP* genes in *Phaseolus vulgaris*, *Arabidopsis thaliana*, *Glycine max*, *Oryza sativa*, and *Zea mays.* Gray lines indicate the collinear blocks between two species, and blue lines indicate syntenic *TLP* gene pairs.

To clarify the evolutionary relationships and phylogenetic characteristics of *TLP* genes, this study constructed synteny diagrams comparing the common bean with other plants, including two dicotyledonous plants (*Arabidopsis* and soybean) and two monocotyledonous plants (rice and maize, Figure 4). The synteny patterns revealed the following syntenic gene pairs: 38 pairs between 10 *PvTLP* genes and 19 *GmTLP* genes, 9 pairs between 8 *PvTLP* genes and 6 *AtTLP* genes, 8 pairs between 5 *PvTLP* genes and 7 *OsTLP* genes, and only 5 pairs between 4 *PvTLP* genes and 4 *ZmTLP* genes. Compared with monocotyledonous plants, the common bean presented stronger synteny with dicotyledonous plants. The highest level of synteny was observed between the common bean and soybean. These findings indicate that *PvTLPs* and *GmTLPs* exhibit a more closed evolutionary conservation than other plants do.

### 2.6. Analysis of Cis-Acting Elements in the PvTLP Promoter

**Figure 5:**
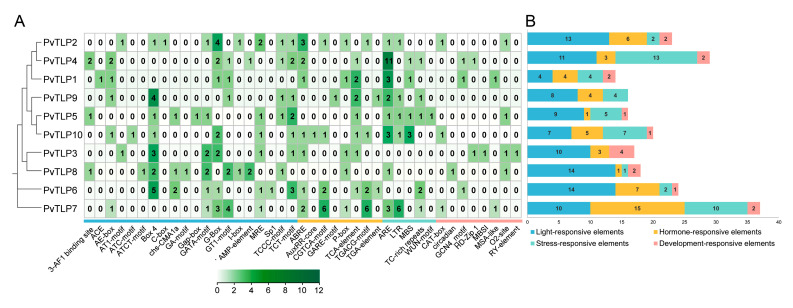
The number of cis-acting elements in the putative promoters of *PvTLP* genes: (**A**) heatmap of the number of cis-acting elements. The color scale represents the abundance of the putative cis-acting elements ranging from white to dark green. The larger the number, the darker the green color. The count of cis-acting elements is marked in the box; (**B**) the stacked bar chart visualizes the statistics of cis-acting elements of each gene in four types. The colors blue, orange, green, and pink indicate the light-, hormone-, stress-, and development-responsive elements, respectively.

To elucidate the potential functions and environmental response mechanisms of the *PvTLP* gene family in the common bean, we extracted 2000 bp upstream sequences from the start codons of 10 *PvTLP* genes and analyzed the cis-acting elements using the PlantCARE database. A total of 41 distinct cis-acting elements were identified and classified into four functional categories: light-, hormone-, stress-, and development-responsive elements (Figure 5). All *PvTLP* genes, except for *PvTLP3* and *PvTLP9*, contained the four categories of functional elements. Light-responsive elements, including Box 4, G-Box, and GT1-motif, are widely distributed across all *PvTLP* promoters. There are five types of hormone-responsive elements: abscisic acid-responsive elements (ABRE) in seven genes, MeJA-responsive elements (CGTCA-motif and TGACG-motif) in four genes, gibberellin-responsive elements (GARE-motif and P-box) in six genes, salicylic acid-responsive elements (TCA-element) in seven genes, and auxin-responsive elements (AuxRR-core and TGA-element) in three genes. Additionally, abundant stress-responsive elements, such as anaerobic induction element (ARE), low-temperature response element (LTR), drought response element (MBS), and defence and stress-responsive element (TC-rich repeats), were identified in the promoter regions. With the exception of *PvTLP9*, one to four development-responsive elements were detected in all *PvTLP* promoters. These included elements related to the cell cycle (MSA-like), palisade mesophyll cells (HD-Zip 1), and Zein metabolism regulation element (O2-site). These results indicate that *PvTLP* genes may play a major role in regulatory pathways and biological processes through complex transcriptional networks.

### 2.7. Expression Patterns of PvTLP Genes in Different Tissues

**Figure 6:**
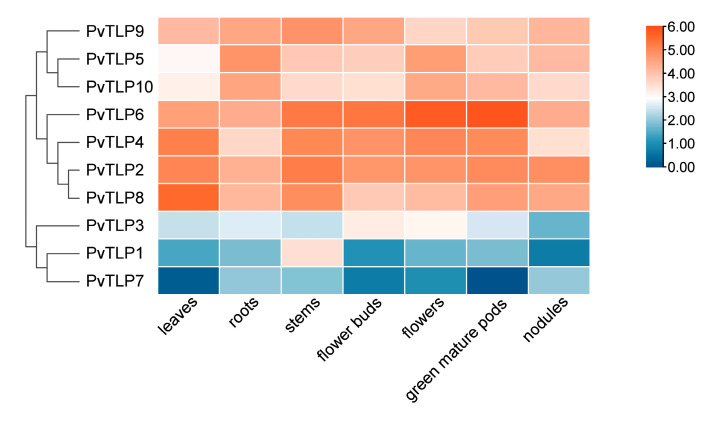
Heatmap of *PvTLP* gene expression profiles in various tissues, including leaves, roots, stems, flower buds, flowers, green mature pods, and nodules. The color scale represents the FPKM values normalized by log2. Orange indicates high expression levels and blue indicates lower expression levels. FPKM, Fragments Per Kilobase of exon model per Million mapped fragments.

Using transcriptome sequencing data from the Phytozome database (Supplementary Table S3), we analyzed the expression levels of *PvTLP* genes in various tissues, including leaves, roots, stems, flower buds, flowers, green mature pods, and nodules. The analysis revealed significant differences in the expression patterns of *PvTLP* genes in these tissues (Figure 6). The expression profiles of *PvTLP* genes were categorized into two groups: Group I, which included seven genes (*PvTLP2*, *PvTLP4*, *PvTLP5*, *PvTLP6*, *PvTLP8*, *PvTLP9*, and *PvTLP10*), exhibited widespread expression in all tissues. In contrast, Group II, comprising three genes (*PvTLP1*, *PvTLP3*, and *PvTLP7*), presented specific expression in certain tissues. Notably, *PvTLP6* was expressed at higher levels in flowers and green mature pods than in other tissues, whereas *PvTLP1* was expressed at a relatively higher level in stems compared with other tissues. Additionally, the segmentally duplicated gene pair *PvTLP2* and *PvTLP4* presented similar expression levels in above-ground tissues. These results suggest that *PvTLP* genes are involved in tissue development and growth in the common bean.

### 2.8. Expression Patterns of PvTLP Genes Under Salt and Drought Stresses

**Figure 7:**
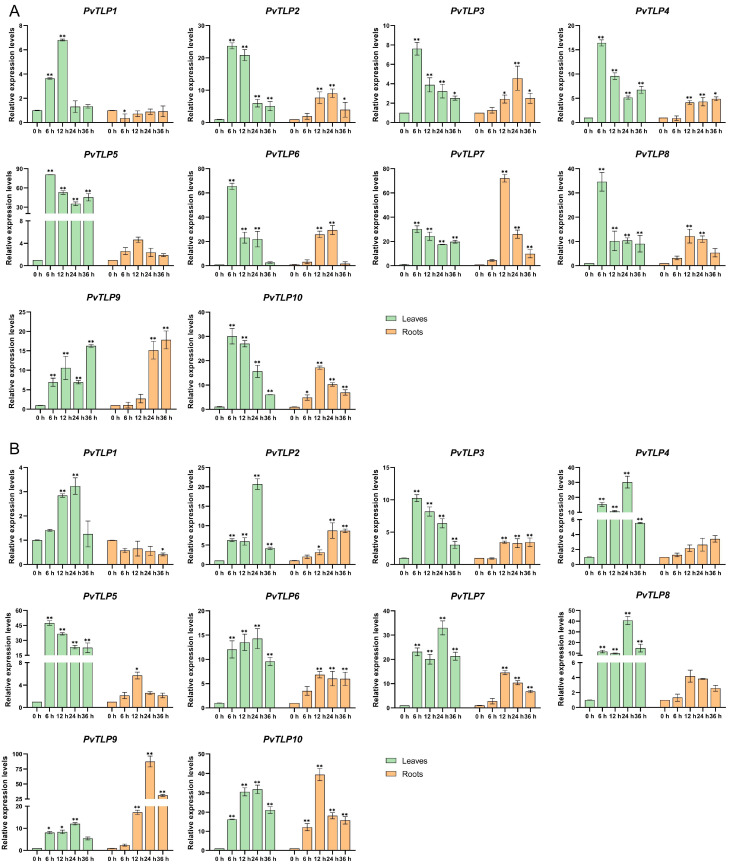
Expression patterns of *PvTLP* genes in the leaves and roots under salt and drought stresses by qRT-PCR analysis: (**A**) expression patterns of *PvTLP* genes under 200 mmol L^−1^ NaCl treatment; (**B**) expression patterns of *PvTLP* genes under 20% PEG6000 treatment. Data were normalized using the common bean *actin* gene. Error bars represent ±SD of three biological replicates. Asterisks indicate statistically significant differences (*, *p* < 0.05; **, *p* < 0.01), determined by the two-way ANOVA statistical test.

Previous studies have confirmed that *TLP* genes respond to various abiotic stresses. To further validate this mechanism, we analyzed the expression patterns of 10 *PvTLP* genes in the leaves and roots of the common bean under salt and drought stresses using qRT-PCR (Figure 7). Our findings revealed various changes in the expression levels of these genes in response to both types of stresses. Under salt stress, the expression levels of eight genes (*PvTLP2*, *PvTLP3*, *PvTLP4*, *PvTLP5*, *PvTLP6*, *PvTLP7*, *PvTLP8*, and *PvTLP10*) in the leaves peaked at 6 h after treatment, with *PvTLP5* showing the greatest induction (81-fold greater than that of the control), followed by gradual downregulation. In the roots, the expression levels of three genes (*PvTLP7*, *PvTLP8,* and *PvTLP10*) exhibited a highly significant increase at 12 h, whereas the other three genes (*PvTLP2*, *PvTLP3*, and *PvTLP6*) peaked at 24 h. Under drought stress, the expression levels of eight genes (*PvTLP1*, *PvTLP2*, *PvTLP4*, *PvTLP6*, *PvTLP7*, *PvTLP8*, *PvTLP9*, and *PvTLP10*) in the leaves increased to a peak at 24 h, whereas five genes (*PvTLP3*, *PvTLP5, PvTLP6, PvTLP7*, and *PvTLP10*) in the roots reached their peak significantly at 12 h. Additionally, most *PvTLP* genes tended to exhibit low/high/low expression patterns under drought stress. These findings imply that *PvTLP* genes may be essential for the response of the common bean to salt and drought stresses.

## 3. Discussion

*TLP* genes play critical roles in plant biological processes, including growth and development, signal transduction, and stress responses. In this study, we identified 10 *PvTLP* genes in the common bean through a comprehensive genome-wide analysis. The number of *PvTLP* genes is significantly lower than that in *Triticum aestivum* (40) and *Brassica napus* (29), and slightly lower than that in *Salvia miltiorrhiza* (12), *Brachypodium distachyon* (12), *Cucumis sativus* (11), and *Arabidopsis thaliana* (11), but greater than that in *Malus domestica* (9) and *Fragaria vesca* (8) [14,19,20,33,34,35,36,37]. The subcellular localization of plant TLPs is diverse. In tomato, 8 out of 11 SlTLP-GFP fusion proteins were found in the nucleus, 2 were detected in both the nucleus and plasma membrane, and 1 was located in the plasma membrane [18]. In addition, the AtTLP5-GFP fusion protein was observed in both the cytosol and plasma membrane [24]. The results of computational predictions concerning the subcellular localization suggested diverse distribution patterns of 10 PvTLP proteins. This diversity may be attributed to different domains or regulatory mechanisms. Therefore, it is essential to conduct further subcellular localization experiments involving PvTLPs fused with GFPs to confirm these findings and gain a deeper understanding of their roles.

Phylogenetic analysis has clarified evolutionary relationships within the *TLP* gene family in the common bean and other plants. To investigate these relationships, a phylogenetic tree of 69 full-length TLP proteins from the common bean, *Arabidopsis*, soybean, rice, and maize was constructed. The results demonstrated their classification into three subfamilies (A, B, and C, Figure 2). The distribution of TLPs across all subfamilies in the five species suggested that the TLPs might have derived from an ancient common ancestor before the divergence of monocotyledons and dicotyledons. The number of PvTLPs in subfamily A was much greater than that in subfamilies B and C, which was consistent with findings related to poplar and wheat [15,33]. Subfamily A was further divided into three subgroups (A1–A3), suggesting the importance of the functional diversification of TLPs. Subfamilies A and B contained both tubby and F-box domains, whereas subfamily C lacked the F-box domain (Figure 3B), likely because the F-box domain was lost during the long-term evolutionary process. Furthermore, the differences in intron number among eukaryotic species are closely associated with the functional diversity of genes [38,39]. Intron/exon analysis revealed that subfamilies A and B of *PvTLP* genes contained three to four introns (Figure 3A), which is similar to the findings of previous studies in rice and strawberry [15,20]. In contrast, subfamily C presented a notable increase in the number of introns, such as *AtTLP8*, *OsTLP4*, *MdTLP8*, *MdTLP9*, and *FvTLP8*, which contained eight, seven, seven, nine, and eight introns, respectively [14,15,19,20]. In the common bean, *PvTLP7* had eight introns (Figure 3A). The high number of introns in subfamily C suggests that these genes may be involved in specific biological functions. Notably, at least one member belonging to subfamily C has been identified in the *TLP* gene family of different plants, indicating that subfamily C is a relatively unique cluster that may perform critical biological functions in plant development. Significant structural and domain-based distinction among subfamilies verifies the validity of the phylogenetic classification of the *TLP* gene family. Furthermore, the distribution of the motifs identified by the MEME suite demonstrates conservation among PvTLP proteins. According to the phylogenetic tree, gene structure, and motif analysis, members of the same subfamily presented similar gene structures and conserved motifs.

Gene duplication events significantly contributed to the rapid expansion of plant gene families. One consequence of gene duplication is the generation of genetic redundancy [40]. In the *Arabidopsis* genome, gene duplication events are widespread. Most mutants with a single loss-of-function mutation do not produce obvious phenotypic effects, which may be due to functional compensation resulting from gene redundancy [41]. Previous reports have shown that *WRKY54* and *WRKY70*, as a pair of duplicated genes, both act as negative regulators of leaf senescence [42]. The genes presented similar expression patterns during leaf development. Mutants of *wrky54* or *wrky70* displayed no obvious or only a weak phenotype of premature senescence, respectively. However, compared to the single mutants, the *wrky54*/*wrky70* double mutants presented a pronounced early senescence phenotype. These findings indicate that *WRKY54* and *WRKY70* present functional redundancy in regulating leaf senescence [43]. In the common bean, a segmentally duplicated gene pair (*PvTLP2*/*PvTLP4*) has been identified. The identity of these proteins was 78.74%, and they exhibited similar expression patterns in above-ground tissues. This finding suggests their potential functional redundancy, and they may cooperate to participate in similar regulatory pathways related to plant growth.

Analysis of cis-acting elements in the promoters of different gene families can elucidate their potential biological functions [44]. This study revealed that the promoters of *PvTLP* genes contained four types of cis-acting elements: light-responsive, hormone-responsive, stress-responsive, and development-responsive elements (Figure 5). Light-responsive elements, such as Box 4, GATA-motif, and G-box, accounted for 46.73% of the total cis-acting elements in the *PvTLP* promoters. In *Mahonia bealei*, the GATA-motif in the *ndhF* promoter might suppress benzylisoquinoline alkaloid biosynthesis during the dark/light transitions by downregulating *ndh* expression [45]. Moreover, the G-box interacts with G-box binding factor (GBF1) to regulate blue light-induced photomorphogenic growth in cotyledons in *Arabidopsis* [46]. Light-regulated GBFs translocated from the cytoplasm to the nucleus, suggesting their importance in coordinating light transduction pathways with light-dependent gene expression [47]. The presence of multiple light-responsive elements in all *PvTLP* promoters indicates that the *PvTLP* gene family may play a significant role in light response regulation. Furthermore, the promoters contain various hormone- and stress-responsive elements. AREBs and AREs were widespread in most *PvTLP* genes, which is consistent with research on wheat [33]. These findings suggest that *PvTLP* genes may regulate hormone and abiotic stress responses in the common bean. Notably, the *PvTLP7* promoter had the greatest number of cis-acting elements, including 15 hormone-, 10 light-, 10 stress-, and 2 development-responsive elements. In addition, considering that PvTLP7 has a unique gene structure and conserved motifs, it suggests that PvTLP7 may play a critical role in plant development and stress adaptation, but more experimental evidence is needed.

Plant growth and development are regulated by complex gene regulatory networks and signaling pathways. Expression profiles have revealed the dynamic regulatory mechanisms of *TLP* genes. In tomato, *SlTLP1* and *SlTLP2* are highly exhibited throughout fruit development, with experimental evidence suggesting their involvement in ethylene-dependent fruit ripening pathways [48]. *AtTLP6* and *AtTLP7* presented pollen-specific expression, where the *attlp6* mutants produced 5–10% aborted pollen, 15% pollen with aberrant male germ units, and 5% bicellular pollen. Similarly, *attlp7* mutants displayed 20–25% pollen with disorganized male germ units and 5–10% bicellular pollen due to cytoskeleton dysfunction [24,49]. Heatmap analysis revealed that 10 *PvTLP* genes presented constitutive or tissue-specific expression patterns (Figure 6). For example, *PvTLP6* was significantly expressed in flowers and green mature pods, indicating its regulatory role in the process of floral-to-pod development. These findings provide a theoretical foundation for the functional characterization of the *PvTLP* gene family.

Abiotic stress seriously threatens crop yields by impairing essential physiological and biochemical processes. Research has verified that *TLP* genes play multiple functional roles in enhancing crop tolerance to abiotic stress such as drought and salinity. In foxtail millet, most *SiTLP* genes were regulated by salt and high-temperature conditions [50]. The expression patterns of apple *MdTLP* genes significantly differed under various abiotic stresses and hormone treatments, with *MdTLP4* and *MdTLP7* consistently upregulated in response to all stresses [19,51,52]. In *Arabidopsis*, *MdTLP7*-overexpressing seedlings presented improved tolerance to cold, heat, osmotic, and salt stresses [53]. Through qRT-PCR analysis, the expression levels of most *PvTLP* genes exhibited significant changes in both leaves and roots after salt and drought stresses (Figure 7). In the leaves, the expression of eight *PvTLP* genes, excluding *PvTLP1* and *PvTLP9*, peaked at 6 h under salt stress. Nevertheless, eight *PvTLP* genes reached their maximum expression levels at 24 h under drought stress, suggesting that *PvTLP* genes in the leaves respond more rapidly to salt stress. In the roots, most *PvTLP* genes exhibited peak expression at 12–24 h after both treatments. Notably, *PvTLP5* expression levels in the leaves rapidly increased at 6 h under both stresses, with expression levels reaching 81-fold greater than that of the control after salt treatment and 48-fold greater than that of the control after drought treatment. The presence of multiple stress-responsive elements in the promoter regions of *PvTLP5* further indicates that *PvTLP* genes may be essential for adapting to abiotic stress in the common bean. Therefore, *PvTLP5* may be a promising candidate gene for genetic improvement to increase stress resistance. The results suggest that *PvTLP* genes respond to both salt and drought stresses. However, these findings are based on gene expression levels, and the exact mechanism by which *PvTLP* genes operate in the regulatory pathway remains unclear. Based on the excellent genes identified in this study, molecular biology techniques will be further employed for plant transformation (such as gene overexpression or knockout experiments) and functional verification, to elucidate the specific functions of *PvTLP* genes and the mechanisms involved in response to environmental stress. This study provides an important theoretical foundation for understanding the functions of *PvTLP* genes and developing breeding strategies to increase stress tolerance in the common bean.

## 4. Materials and Methods

### 4.1. Genome-Wide Identification of PvTLP Genes

The genome data and gene annotation files of the common bean (v2.1) were downloaded from the Phytozome database (http://phytozome.jgi.doe.gov/, accessed on 18 February 2025), and *Arabidopsis* AtTLP protein sequences were obtained from the TAIR database (https://www.arabidopsis.org/, accessed on 18 February 2025). To identify the candidate TLP proteins, AtTLP protein sequences were used as queries in a local BLASTP alignment [54]. For further validation, the Hidden Markov Model (HMM) of the tubby domain (PF01167) was retrieved from the Pfam database (http://pfam.xfam.org/, accessed on 20 February 2025) and analyzed using HMMER 3.3 with the default parameters [55,56]. All proteins of the putative TLP proteins were subsequently subjected to batch CD-search (https://www.ncbi.nlm.nih.gov/cdd, accessed on 20 February 2025) to confirm the presence of complete tubby domains [57]. The physicochemical properties of the TLPs were predicted using the ExPASy online server (http://web.expasy.org/protparam/, accessed on 22 February 2025) [58]. Subcellular localization analysis was conducted using the WoLF PSORT online server (https://www.genscript.com/wolf-psort.html/, accessed on 24 February 2025) [59].

### 4.2. Chromosomal Localization and Gene Replication Events

According to the common bean genome data and annotation information, TBtools-II (version 2.156) was employed to visualize the chromosomal localization map for 10 *PvTLP* genes [60]. The synteny analysis of *TLP* genes was conducted using the MCScanX software [61]. The ratio of the non-synonymous substitution rate (Ka) to the synonymous substitution rate (Ks) for the duplicated *PvTLP* gene pairs was calculated using the TBtools-II software. A Ka/Ks ratio greater than 1 indicates positive selection, a ratio of 1 indicates neutral selection, and a ratio less than 1 indicates purifying selection or negative selection.

### 4.3. Multiple Sequence Alignment and Phylogenetic Analysis

Full-length protein sequences of 10 PvTLPs, 11 AtTLPs, 20 GmTLPs, 14 OsTLPs, and 14 ZmTLPs were retrieved from previous studies (Supplementary Table S2) [14,15,17,62]. Previous research identified 15 *ZmTLP* genes in maize; however, we found that *ZmTLP15* is a non-translating gene. Multiple sequence alignment wa s performed on the 69 TLP protein sequences using Muscle. The neighbor-joining (NJ) method was employed to construct the phylogenetic tree in the MEGA11 software with 1000 bootstrap replicates [63,64]. iTOl (https://itol.embl.de/, accessed on 9 March 2025) was used for visualization and beautification [65].

### 4.4. Gene Structure, Conserved Domain, and Motif Analysis

A batch CD-search (https://www.ncbi.nlm.nih.gov/cdd, accessed on 22 February 2025) was performed to identify the conserved domains of PvTLP proteins. The PvTLP protein sequences were submitted to the MEME online software (https://meme-suite.org/meme/, accessed on 22 February 2025) to predict the conserved motifs, with the maximum number of motifs configured to 10 [66]. Gene structure, conserved domain, and motif were integrated and visualized using TBtools-II.

### 4.5. Synteny Analysis

The genomic sequences and annotation information for *Arabidopsis* and soybean were downloaded from the TAIR (https://www.arabidopsis.org/, accessed on 18 February 2025) and Phytozome (http://phytozome.jgi.doe.gov/, accessed on 26 February 2025) databases, respectively. Genomic resources and annotation files for rice and maize were retrieved from Ensembl Plants (https://plants.ensembl.org/index.html, accessed on 26 February 2025). We analyzed the inter-species synteny using MCScanX and visualized the results using TBtools-II.

### 4.6. Promoter Cis-Acting Elements Analysis

The 2000 bp upstream sequences of 10 *PvTLP* genes in the common bean were extracted as promoter regions. Subsequently, these sequences were uploaded to the PlantCARE database (https://bioinformatics.psb.ugent.be/webtools/plantcare/html/, accessed on 28 February 2025) to cis-acting elements prediction [67]. After screening and classification, the main cis-acting elements related to light, hormone, stress, and development were identified. Data visualization was performed with TBtools-II and Excel 2021.

### 4.7. Heatmap Analysis

The transcriptome sequencing data for *PvTLP* genes in various tissues were retrieved from the Phytozome database (http://phytozome.jgi.doe.gov/, accessed on 25 February 2025, Supplementary Table S3). Expression levels were determined using FPKM (Fragments Per Kilobase of exon model per Million mapped fragments) values and normalized using the log2 (Fold Change) transformation. Expression profiles were generated using the heatmap function from OmicShare tools (https://www.omicshare.com/tools/, accessed on 1 March 2025) [68].

### 4.8. Plant Material Source and Treatments

The common bean cultivar Pinjinyun 4 is a bred variety from Shanxi, China. The seedlings were grown until the trifoliate leaves had fully expanded in an artificial climate chamber at 25 °C, with a 16 h light/8 h dark photoperiod and 70% relative humidity. For salt treatment, the seedlings were cultured in Hoagland solution supplemented with 0 or 200 mmol L^−1^ NaCl. For drought treatment, the seedlings were cultured in Hoagland solution supplemented with 0 or 20% PEG6000. Leaves and roots were collected at 0, 6, 12, 24, and 36 h after NaCl and PEG6000 treatments, frozen quickly in liquid nitrogen, and stored at −80 °C.

### 4.9. RNA Extraction and qRT-PCR Analysis

Total RNA was extracted from the leaves and roots of the common bean using the RNA Easy Fast Plant Tissue Kit (Tiangen, Beijing, China). The concentration and purity of the RNA were assessed using a NanoDrop 2000 spectrophotometer (Thermo Fisher Scientific, Waltham, MA, USA), and the quality of the RNA was confirmed by 1% agarose gel electrophoresis. Total RNA was reverse-transcribed into cDNA using the PrimeScript Fast RT reagent Kit with gDNA Eraser (Takara, Otsu, Japan). qRT-PCR was conducted on an Applied Biosystems QuantStudio 6 Flex cycler (Applied Biosystems, Waltham, MA, USA) following the protocol of the SYBR Premix Ex Taq Kit (Takara Bio, Otsu, Japan): initial denaturation at 95 °C for 30 s, followed by 40 cycles of 95 °C for 5 s, and 60 °C for 34 s. Each experiment included three technical replicates and three biological replicates. The common bean *actin* gene (Phvul.011G064500) was used as the internal control. Relative quantification was performed using the 2^−ΔΔCt^ method [69]. Statistically significant differences were determined by two-way ANOVA followed by Dunnett’s multiple comparisons test and data visualization, which were performed using GraphPad Prism version 8.4.0 (Boston, MA, USA, www.graphpad.com). Primer sequences are provided in Supplementary Table S4.

## Figures and Tables

**Table 1:** Detailed information of the *PvTLP* gene family.

Gene Name	Gene ID	CDS (bp)	Protein (aa ^1^)	Intron	MW ^2^ (Da)	pI ^3^	Instability Index	Aliphatic Index	GRAVY ^4^	Subcellular Localization ^5^
*PvTLP1*	Phvul.001G013500	1290	429	3	47,795.75	9.34	60.34	80.93	−0.341	Nucl
*PvTLP2*	Phvul.002G031500	1245	414	3	46,047.76	9.46	56.58	80.05	−0.346	Nucl
*PvTLP3*	Phvul.003G100700	1083	360	4	40,469.27	9.09	57.71	79.67	−0.302	Nucl
*PvTLP4*	Phvul.003G254900	1242	413	3	46,210.86	9.21	61.47	77.92	−0.334	Chlo
*PvTLP5*	Phvul.004G095000	1278	425	3	47,566.43	9.56	67.49	76.40	−0.371	Nucl
*PvTLP6*	Phvul.005G082200	1128	375	3	41,640.27	9.33	53.17	67.09	−0.476	Mito
*PvTLP7*	Phvul.005G183800	1113	370	8	42,252.31	9.60	37.45	65.62	−0.607	Nucl
*PvTLP8*	Phvul.006G161100	1152	383	4	43,206.85	9.46	60.29	76.37	−0.327	Mito
*PvTLP9*	Phvul.006G210500	1284	427	3	47,950.04	9.47	63.82	76.28	−0.363	Nucl
*PvTLP10*	Phvul.007G079900	1296	431	3	48,120.22	9.71	60.90	75.31	−0.349	Nucl

^1^ aa: the number of amino acid residues; ^2^ MW: molecular weight; ^3^ pI: theoretical isoelectric point; ^4^ GRAVY: grand average of hydropathicity; ^5^ Subcellular Localization: Nucl: nucleus, Chlo: chloroplast, Mito: mitochondria.

## Data Availability

All the data that support the findings of this study are available in the paper and its Supplementary Materials published online.

## Supplementary Materials

The following supporting information can be downloaded at https://www.mdpi.com/article/10.3390/ijms26125702/s1.
