# Supplementary material for: Characterization and Expression Analysis of the PvTLP Gene Family in the Common Bean (Phaseolus vulgaris) in Response to Salt and Drought Stresses
Source: Int J Mol Sci. 2025 Jun 13;26(12):5702. doi: 10.3390/ijms26125702 (PMC12193164; doi:10.3390/ijms26125702)
Supplement: Supplementary file 1 [file ijms-26-05702-s001.zip › ijms-3599600-supplementary.pdf]

Table S1. Primer sequences of *PvTLP* genes for qRT-PCR..

Primer Name	Primer Sequence
TLP1-F	5′-AGAGGGAAAGCATGTGCATCGC-3′
TLP1-R	5′-TGAGGAGGAGGAGGAGGAGGATG-3′
TLP2-F	5′-GTGGAGAGCAGGCGTTGGAATG-3′
TLP2-R	5′-GCCCACTGCCCTTGCTGAATAG-3′
TLP3-F	5′-CGTGCTGTTGTTGTCTTCTGTGC-3′
TLP3-R	5′-GTGGACCGGGCTGCTTCAATG-3′
TLP4-F	5′-GATGGGTTGGAGCAGAGTTGGTG-3′
TLP4-R	5′-CTTGGCCTCCGACGACTCAATTC-3′
TLP5-F	5′-AGCACCACGATGGCATGAACAG-3′
TLP5-R	5′-TGATGACGGCGGTGGAGGAG-3′
TLP6-F	5′-GCTCGGATTTTCTTGGCACG-3′
TLP6-R	5′-ACCTGCCCCACCTCAAAGT-3′
TLP7-F	5′-CGAACCGCCAGAACGTAGTGTC-3′
TLP7-R	5′-GGGCTGTTTAAGGCACGAAGGG-3′
TLP8-F	5′-ACAGCCTGGTCCAAGGGAGAATC-3′
TLP8-R	5′-TTGCGTGCAGCCAGAAGGAAC-3′
TLP9-F	5′-TGCTGCCGTTTGCCAGTCTTG-3′
TLP9-R	5′-CGTGGTCCAGGCTGCTTCAAG-3′
TLP10-F	5′-GCAGCCAGCGGTCATACAGAAC-3′
TLP10-R	5′-ACGACCAGGCCATGTACTCTCAC-3′
Actin-F	5′-GAAGTTCTCTTCCAACCATCC-3′
Actin-R	5′-TTTCCTTGCTCATTCTGTCCG-3′
